# Rhizarthrosis Part II: A New Approach of Manual Therapy and Therapeutic Exercise

**DOI:** 10.7759/cureus.52999

**Published:** 2024-01-26

**Authors:** Saverio Colonna, Corrado Borghi

**Affiliations:** 1 Osteopathic Spine Center Education, Spine Center, Bologna, ITA

**Keywords:** rhizarthrosis, mobilization with movement, trapezium-metacarpal joint, manual therapy, mobilization, therapeutic exercise, dynamic stability, biomechanics

## Abstract

Rhizarthrosis (RA), also known as trapezium-metacarpal osteoarthritis, is a degenerative condition affecting the thumb's first joint, leading to functional impairment and pain. Conservative treatment options are preferred for mild to moderate cases (Eaton-Littler grades I and II) and typically encompass a range of therapeutic modalities, including manual therapy. However, for the existing manual therapy techniques, there is a lack of comparative studies for efficacy, and therapeutic exercises are often generic and non-specific to RA.

This study proposes a novel treatment protocol that combines manual therapy with specific therapeutic exercises grounded in the biomechanical analysis of the trapeziometacarpal joint. The focus is on enhancing joint stability, reducing pain, and improving function.

The manual therapy component includes three phases. A passive phase, during which joint distractions are applied to alleviate discomfort and improve joint mobility. An active phase that addresses joint mobility on the adduction-abduction plane, the first plane of movement to suffer limitation: the therapist facilitates the isometric adduction of the thumb, followed by an assisted abduction. A second active phase is where Mulligan's Mobilization With Movement concept is applied. This technique involves passive pain-free joint mobilization with simultaneous active finger movements, to provide additional therapeutic benefits.

The therapeutic exercises component focuses on strengthening the first dorsal interosseous muscle as an abductor to reduce thumb adductor muscle activation and joint stress. Patients are encouraged to perform finger spreading exercises using a rubber band between the first and fifth fingers, emphasizing first dorsal interosseous activation and stability of the thumb. This type of muscle strengthening does not involve movement of the trapeziometacarpal joint. It is recommended to start performing 5-10 repetitions or 5 seconds of isometric contraction, repeat throughout the day, and progressively increase the load by adding a turn to the rubber band or changing it, increasing the number of repetitions bringing it to 15 and/or increase the isometric contraction time to 10/15 seconds.

The proposed therapeutic rationale, informed by biomechanical insights, lays a promising foundation for further investigation. Nevertheless, empirical validation through rigorous clinical trials remains essential to substantiate its clinical utility and advance the management of RA.

## Introduction

Rhizarthrosis (RA), or trapeziometacarpal osteoarthritis, is an arthritic degenerative process that affects the first joint of the thumb, i.e., the one between the trapezius bone and the base of the first metacarpal (trapeziometacarpal joint - TMj). The thumb is responsible for more than 40% of the functions of the hand because the ability to grasp and squeeze is ineffective without its opposability and its prehensile abilities [1]. Therefore, a degeneration of the thumb joint can be highly disabling.

Conservative treatment essentially aims to reduce pain and mechanical load on the joint and relieve inflammation. This results in maintaining or increasing strength, function, stability, and mobility while improving occupational performance. Conservative treatment is preferred to surgery in mild forms at initial stages (I and II grade of Eaton-Littler classification of the disease, before significant TMj destruction; for a more in-depth presentation of the Eaton-Littler scale, see Part I [2,3]). Anyway, the choice of treatment depends more on the severity of the reported symptoms and the functional needs of the patient, than Eaton-Littler's radiographic staging [4]. Conservative treatment involves multiple therapeutic approaches: orthoses, therapeutic exercise, manual therapy, physical therapy, and infiltrative therapy [5].

The authors who published the most on manual therapy applied to RA are Villafañe and colleagues [6-10]. These authors proposed four different manual therapy techniques: neurodynamic [11], Kaltenborn [12], Mulligan [13], and Maitland [14]. A summary of these techniques was presented in Part I [2]. These works, which were produced by the same research group, do not report a comparison of the results between the different techniques used, to verify whether there is one that is better than the others in terms of effectiveness [6-10,15]. Furthermore, these studies do not provide a proposal for a specific therapeutic exercise to be combined with manual treatment. The only study that proposed the therapeutic association between manual therapy and exercise was that of Villafañe and colleagues [15] who utilized an exercise program previously presented by Rogers and Wilder [16]. This exercise program, however, was not specific for the TMj nor for the osteoarthritis of the hand. Instead, a study protocol of specific exercises for RA has been published, but this work did not include the association with manual therapy and awaits experimental verification [17].

The aim of this report is to propose a therapeutic rationale that includes a part of manual therapy and a part of therapeutic exercise deriving from the biomechanical analysis of the functioning of the TMj.

## Technical report

Biomechanics of TMj

**Figure 1:**
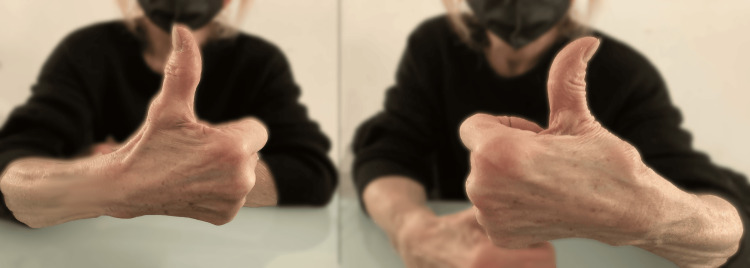
Example of TMj osteoarthritis It is possible to note in the pathological left hand, compared to the right, a limitation of the abduction of the TMj and an increase in the abduction of the first phalanx of the first finger. TMj: trapeziometacarpal joint

The stability of TMj is due to the fine balance between the passive ligament structures and the active muscle-tendon structures (Figure 1). The passive stability of the TMj is mainly determined by five ligaments [5]: anterior oblique divided into superficial and deep (beak ligament); ulnar collateral; inter metacarpal; oblique posterior; and radial collateral. Although there is controversy over the primary passive stabilizers of TMj, several studies have concluded that the anterior oblique ligament and radial collateral, also referred to as dorsal radial, are the primary stabilizers [18,19].

**Figure 2:**
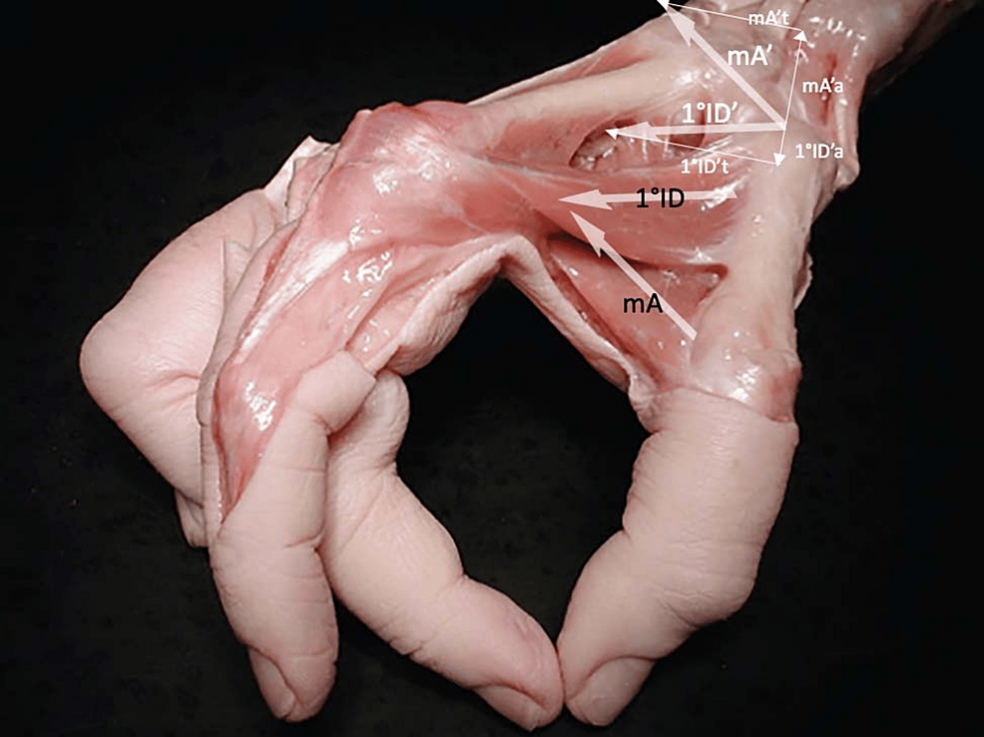
Force vectors of first dorsal interosseous and adductor Force vectors during the grip of the first dorsal interosseous (1st ID in the figure) and the adductor (mA). These vectors translated at the level of the TMj are 1st ID' and mA'. Decomposition of these forces produces the following components: mA't and 1°IDt, which are the transversal components; mA'a and 1°IDa, which are the axial components (modified from Schmidt and Lanz [20]).

The muscles that with their action can affect the TMj are adductor pollicis (oblique and transverse head), short and long flexor (deep and superficial head), short and long abductor, opponent, short and long extensor, firs palmar and dorsal interossei, lumbricals. Active stability, managed by the thumb muscles, has not yet been clarified. The activation of each muscle provides a force vector which, on the one hand, can improve congruity and, on the other, could increase instability. Figure 2 shows the direction of the force vector of the main muscles that act on the TMj.

Regarding the compaction force determined by the muscles during the grip, a dated study by Cooney and Chao showed that during a grip that generates 1 kg at the level of the fingertips, the calculated compression loads were 3 kg at the interphalangeal joint, 5.4 kg at the metacarpal phalangeal joint and 12 kg at the TMj [21].

**Figure 3:**
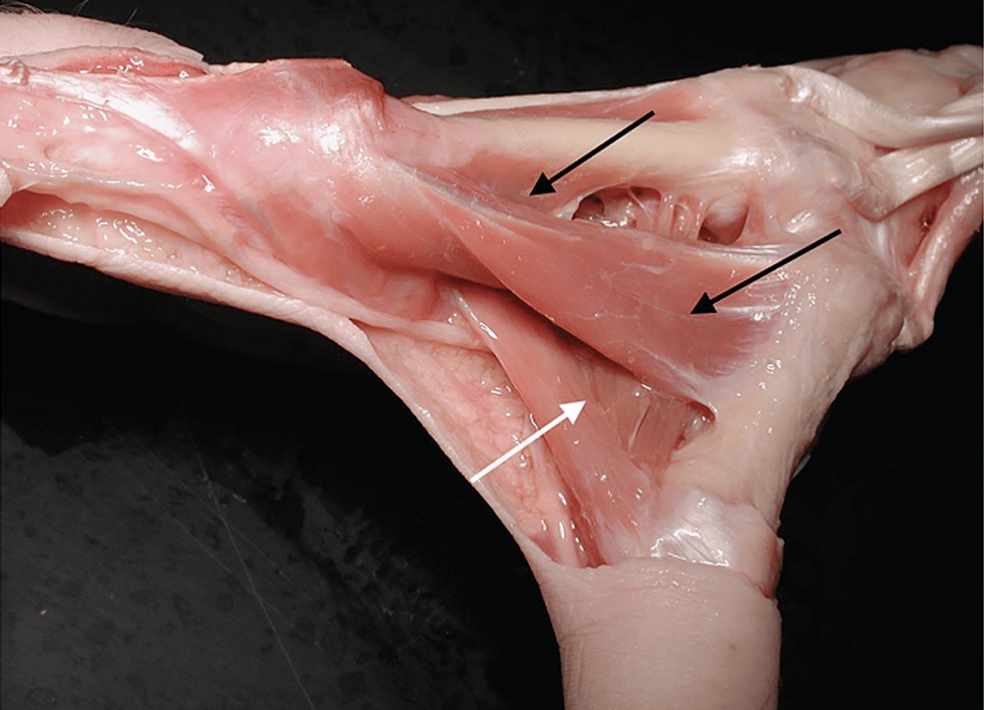
First dorsal interosseous and adductor Image of right-hand anatomical finding, dorsal view, where the 1st DI has been highlighted with the two ends (black arrows). The adductor is indicated with a white arrow. With permission from Schmidt and Lanz [20]. 1st DI: first dorsal interosseous

Biomechanical studies by Brand and Hollister highlighted the importance of the first dorsal interosseous (1st DI) in TMj stability [22]: when in the lateral pinch position with the thumb muscles loaded, if the tension from 1st DI was removed, TMj had a radial subluxation; after the tension of this muscle was restored, the joint repositioned itself correctly. Boutan found that the opponent and the 1st DI have a torque effect on the base of the 1st metacarpus and the latter is a very active thumb muscle during closed kinetic chain prehensile activities [23]. The 1st DI is a muscle with a dual proximal insertion: the radial one occurs on the proximal half of the first metacarpus, and the ulnar one on the second metacarpus (Figure 3). The distal insertion is realized with a single tendon at the base of the proximal phalanx of the second finger. The 1st DI is innervated by the deep branch of the ulnar nerve [24].

Moulton et al. found the TMj to be more congruent, discharging the volar surface of the trapezius, when the metacarpus is positioned at 30° of flexion on the trapezius [25]. In this position, on one hand, the vectors of the oblique head of the adductor muscle and opponent muscle, on the other hand, the vector of the 1st DI, have the same direction (agonists) on the transverse plane (plane orthogonal to the major axis of the metacarpus) in bringing the first metacarpus closer to the second; however, on the main axis of the TMj, they have opposite directions (antagonists). This implies that the action of the oblique head of the adductor and opponent (indirectly also the flexor) leads to radial compression/sliding of the proximal epiphysis (dislocation), while the 1st DI is opposed (Figure 2).

Considering the image of the horse and the rider proposed by Neumann [26] (see Part I of this work [2]), to make it more specific, in order to better understand the imbalance present at the base of the RA, it is necessary to reconsider first the direction of the rider and second the "horse racing gesture". About the direction, the ulnar direction (face facing the 2nd metacarpus) is closer to reality than the radial one; moreover, the equestrian gesture of jumping at obstacles is more suitable to describe RA causes. In this case, the orientation of the saddle is more inclined, and the rider must seek balance. During the jump, the rider needs the stirrups to keep stability (see Part I [2]); in the event of an insufficient stiffness of stirrups, the rider would rotate forward and slide backward. The stirrups are representative of the ligamentous structures. RA, therefore, is often secondary to insufficient ligament structures as proposed in other joints [27].

Laxity, especially of the anterior oblique ligament of the TMj, causes instability of the joint during translational movements [28]. It is believed that, due to this insufficiency of the anterior oblique ligament, the resulting recurrent stresses during the movements of the first column of the thumb are at the basis of the degeneration of the cartilage [29] which begins on the palmar surface and then extend over the dorso-radial one [30,31].

Proposal of a new protocol of manual therapy and muscle strengthening

Manual Treatment

Manipulative treatment is a choice of therapy used individually or combined with other therapies in osteoarticular degeneration, such as gonarthrosis [32].

For RA, some studies reported good results using different manual techniques, but no study has been performed to verify which technique is the best in terms of results [6-10]. For this reason, the manual therapy protocol we propose, divided into three steps, one passive and two active, includes multiple manual techniques.

In particular, Mulligan’s Mobilization With Movement (MWM) concept is applied [13]. This Mulligan’s treatment concept involves the application of manual mobilization to peripheral and vertebral joints. It is a system of manual therapy intervention that combines a sustained manual "gliding" force to a joint with a concurrent physiologic motion of the joint, either actively performed by the patient or passively performed by the therapist. It is hypothesized that alterations in joint position may occur following injury or due to physiological changes secondary to degenerative conditions [33]. This technique aims to promote the restoration of normal joint alignment and arthrokinematics, rather than the stretching of tightened tissues [33]. The active movement chosen is the one that previously produced pain but, if superimposed on better joint alignment, occurs without pain [34,35]. Therefore, MWMs provide a passive pain-free end-range corrective joint glide with an active movement. MVMs have shown promising clinical results such as a reduction of pain and improved range of motion [36,37]. Originally, these effects were thought to occur as a result of correction of the positional faults in the joint [38]. Contemporary understanding suggests underlying neurophysiological mechanisms of MWM [34,39]. Additionally, mechanisms may include a reduction in fear of movement and a placebo [40].

**Figure 4:**
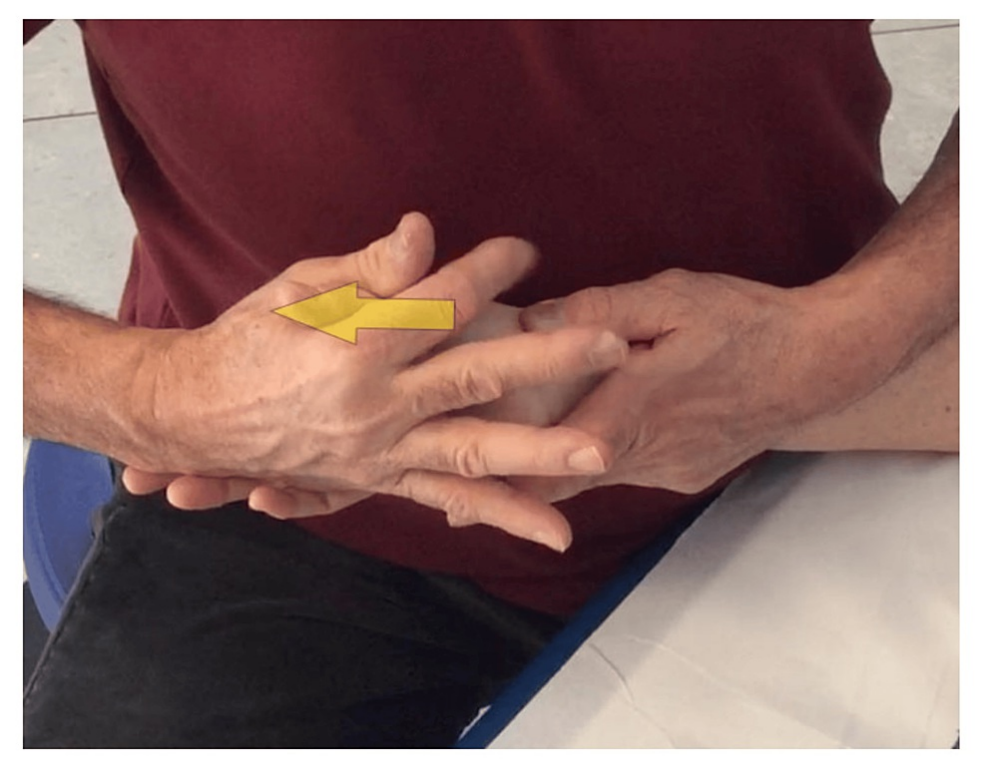
First step (passive phase): TMj decoaptation technique. TMj: trapeziometacarpal joint

First step (passive phase): The patient is lying supine or sitting. The therapist is next to the patient on the pathological side; with hand near the patient, stabilizes the wrist/radius and the thumb stabilizes the trapezius bone; with the thumb and index of the hand on the same side of the pathological hand to be treated, grasp the metacarpal to be treated; with the opposite hand, anchored to the 1st phalanx, he performs grade 3 tractions inducing decoaptation of the TMj (Figure 4). Traction is the technique that distracts one joint surface perpendicular to another, while the gliding technique describes the translational sliding of one joint surface parallel to another [12]. Grade 3 traction was defined as an additional force, which is applied on the parallel axis [41]. This results in the surrounding soft tissue and joint stretching, separating the joint surfaces [12]. Fifteen repetitions are performed of such amplitude and rhythm that they produce at most discomfort or slight pain.

**Figure 5:**
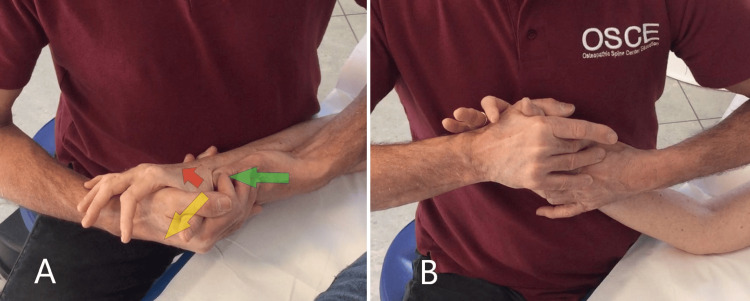
Second step (active phase) Example of treatment for rhizarthrosis of the right thumb: (A) image to show the exact operator finger point of contact/activity; with the thumb of the left hand (green arrow) the operator induces a push to medial/distal on the epiphysis of the 1st metacarpal and with the index finger and thumb of the other hand decoaps and abducts the patient's metacarpal (yellow arrow); having reached the greatest angle of abduction, pain allowed, the patient is asked for isometric adduction/opposition of the 1st metacarpal (red arrow); (B) correct position to use the technique.

Second step (active phase): The patient is lying supine or sitting. The therapist is in the same position as in the previous technique. With the thumb of the hand gripping the wrist, the therapist searches for the joint line of the TMj, contacts the head of the metacarpal, and induces a push in a medial/distal direction (Figure 5A); with the thumb and forefinger the other hand induces, with a leverage effect (forefinger abducting and thumb adducting), abduction of the metacarpal up to an angle permitted by the patient's pain (Figure 5B); at this point, the patient is asked to do isometric adduction of the thumb for about 3-5 seconds; after 3-5 seconds of relaxation, the therapist tries to increase the abduction of the patient's thumb, within the limits of pain, by increasing the combined thrust of both hands. The cycle is repeated 3-5 times. This model of contraction, relaxation, and gain follows the muscle energy techniques (MET) of osteopathy devised by Fred Mitchell Sr. [42]. The objective of this technique is to lengthen the stiffened myofascial structures that maintain the proximal epiphysis of the first metacarpal in an incorrect position.

**Figure 6:**
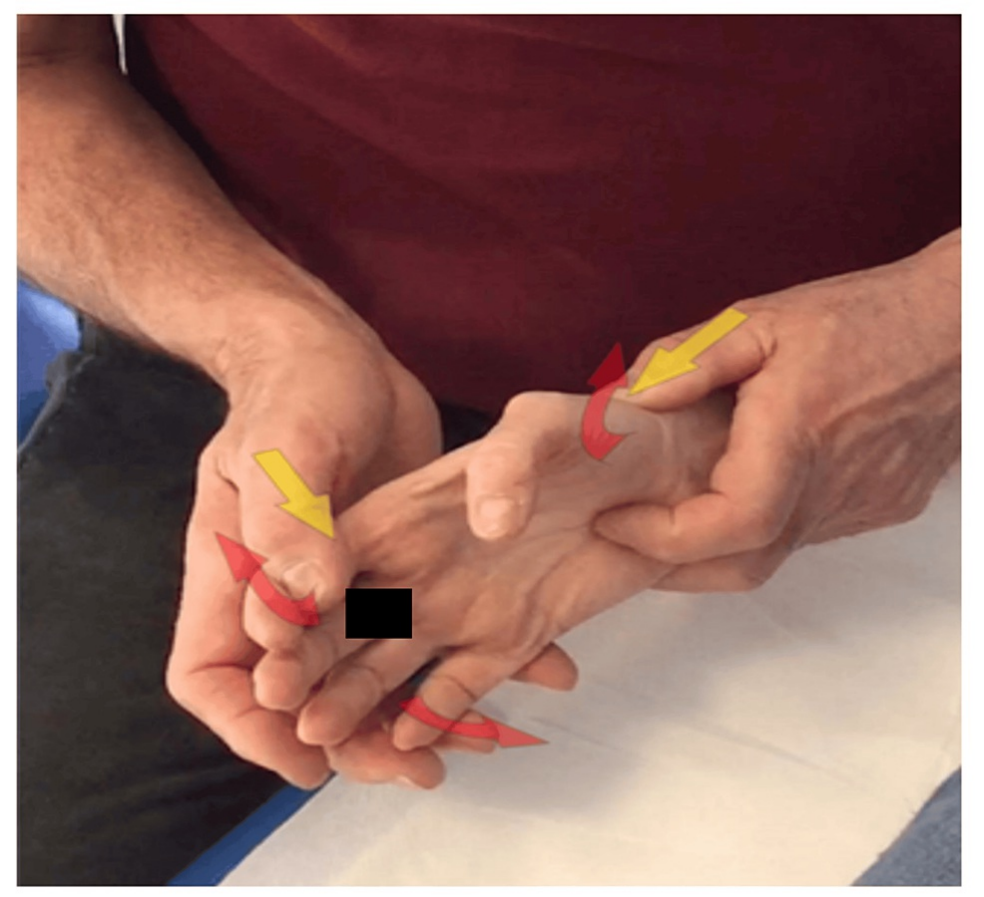
Third step (active phase) Dynamic execution of the abduction according to the Mulligan concept. The yellow arrows indicate the fixed point imposed by the therapist, and the red arrows indicate the movements required with the muscle activation of the patient.

Third step (active phase): The patient is lying supine or sitting; the therapist is in the same position as in the previous technique; with the thumb of the hand gripping the wrist, the therapist searches for the rhyme of the TMj, contacts the head of the metacarpal and induces a push in a medial/distal direction; with the thumb of the other hand, it contacts the radial side of the second finger (Figure 6); at this point, the therapist asks for the maximum opening of the fingers of the hand in a rhythmic way for 15 repetitions or 5 seconds of maximum contractions, followed by 5 seconds of rest repeated 3-5 times. This type of movement involves the activation of the abductors, with relative relaxation of the adductors/flexor/opponents, and simultaneous activation of the first dorsal interosseous (1st DI) in the role of the abductor of the 2nd metacarpal.

Therapeutic Exercises

In recent decades, scientific evidence highlighted how exercise can protect articular cartilage, such as that of the knee, from osteoarthritic degeneration [43]. Physical training aims to improve any function of the body through the patient's own strength, possibly with assisted intervention of the therapist or with equipment support [44]. Regular exercise as well as being able to promote a reduction in pain at a central level can protect chondrocytes against pyroptosis in OA [45,46]. The proposed muscle strengthening is always in line with the fundamental biomechanical role played by the 1st DI muscle [47]. An electromyography (EMG) study of hand muscle use with 1st DI activation found that during dexterity tasks people with RA have weaker muscle strength and take longer to complete tasks [48].

The objective of the exercise we propose is to activate mainly the 1st DI as an abductor, limiting the activation of the thumb adductor muscle. The thumb adductor alone, in fact, generates an altered movement that overloads the capsular ligament structures in stretching and the cartilaginous structures in compression: this phenomenon produces pain, which could lead to muscular inhibition as it happens in other joints of the body [49].

**Figure 7:**
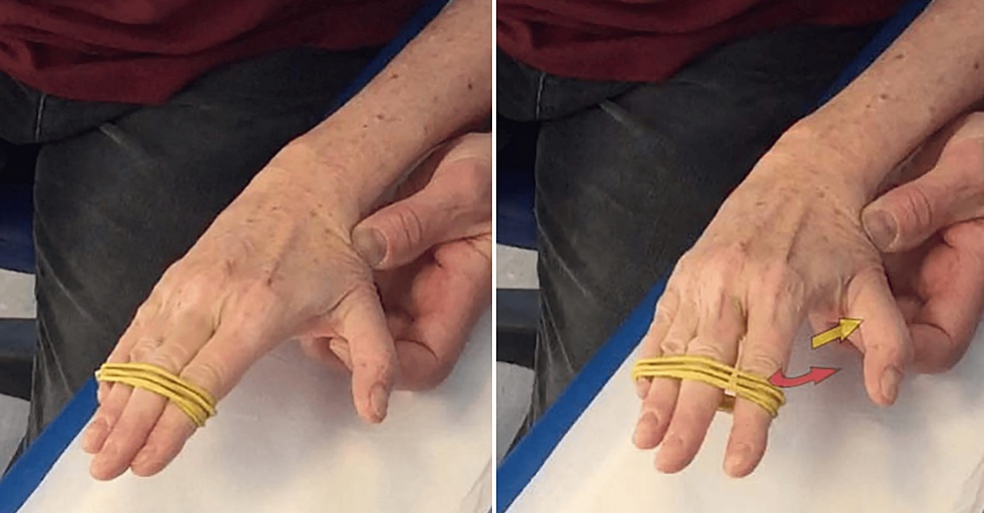
Strengthening of the first dorsal interosseous Technique with a rubber band for selective reinforcement of the 1st DI assisted by the therapist which resists, creating a fixed point that inhibits the movement of the TMj, the adduction of the thumb. 1st DI: first dorsal interosseous

The proposed exercise is easily done using a rubber band stretched between the first and fifth fingers (Figure 7). It is recommended to perform 5-10 repetitions, or 5 seconds of isometric contraction, with maximum spreading of the fingers of the hand. The optimal would be to repeat this type of contraction at least 10 to 15 times a day, observing an adequate period of rest (5-10 min) between one repetition and the next. It is difficult to predetermine the type of rubber band and the force to apply, given the considerable variability of the subjects affected by this pathology. It will be a therapist's task to adequately personalize the intensity of the exercise.

**Figure 8:**
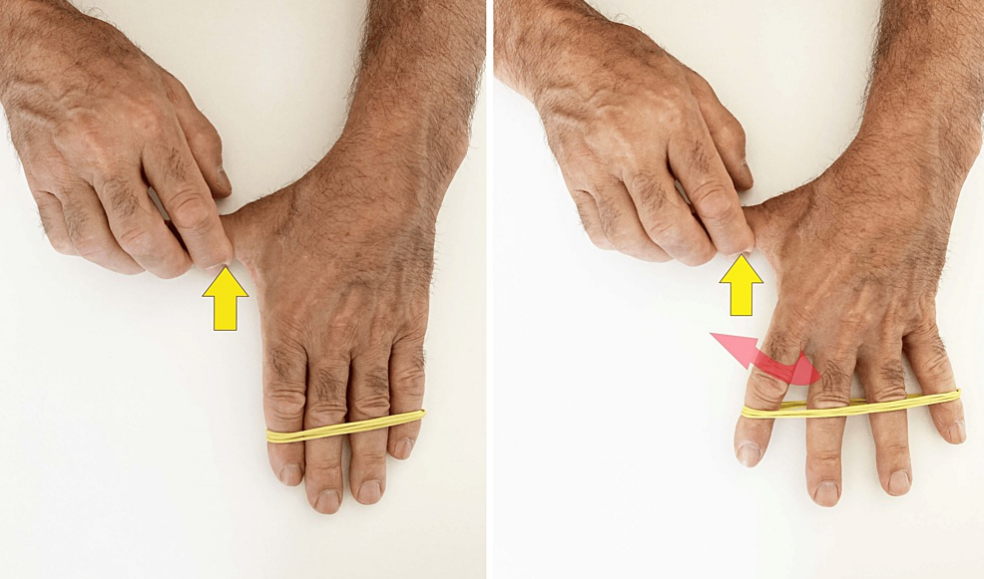
Self-managed strengthening of the first dorsal interosseous - with the other hand. Technique with a rubber band for selective reinforcement of the 1st DI, self-managed by the patient using the other hand to stabilize the first metacarpal. 1st DI: first dorsal interosseous

**Figure 9:**
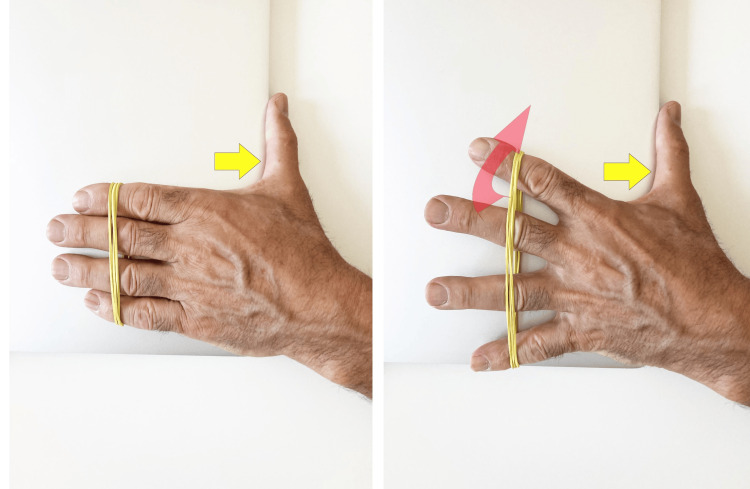
Self-managed strengthening of the first dorsal interosseous - with external stabilization. Technique with a rubber band for selective reinforcement of the 1st DI, self-managed by the patient using a computer edge to stabilize the first metacarpal. 1st DI: first dorsal interosseous

It is important to stabilize the thumb by limiting the TMj adduction so that it acts as a fixed point for the 1st DI: this can be done with the help of the therapist (Figure 7), performed autonomously by the patient with the counter-thrust on the base of the metacarpal of the thumb of the other hand (Figure 8) or using the edge of a computer (Figure 9) or table.

The load should be progressively increased, based on the pain perceived during the exercise. It is possible to increase the load by acting on the rubber band (adding further winding or using another rubber band), on the number of repetitions (bringing them to 15), and/or on the isometric contraction time (up to 10/15 seconds).

## Discussion

The article provides a proposal for the treatment of RA. RA represents a significant challenge in hand pathology due to its impact on thumb joint functionality. Given the potentially debilitating condition caused by a degenerated TMj, the importance of appropriate treatment of RA is well recognized [1-16].

Conservative management stands as the cornerstone in early-stage RA, aligning with the preference to mitigate symptoms and improve functionality before considering surgical intervention [3-5]. However, relevant lacuna within the literature were recognized: a lack of comparative analyses among manual therapy techniques and the absence of specific, targeted therapeutic exercises tailored for TMj rehabilitation [6-10].

It has been highlighted in the literature that constant therapeutic exercise performed for osteoarthritic problems can improve pain [45], protect chondrocytes [46], reduce fear of movement [40], and correct the altered position of the articular surfaces [38].

TMj biomechanics is a fundamental base for appropriate treatment, elucidating the delicate equilibrium between passive ligaments and active muscle structures governing joint stability. Of particular note is the role of the anterior oblique and radial collateral ligaments as pivotal stabilizers [18,19], and the active role played by 1st DI and adductor [17,22-25].

The proposed therapeutic protocol represents a novel integration of manual therapy and exercises grounded in biomechanical principles. The graduated steps of manual treatment, incorporating graded tractions, active phase maneuvers, and Mulligan's MWM concept, signify a promising approach toward addressing TMj stability and function. Noteworthy is the emphasis on activating the 1st DI muscle to mitigate aberrant movements, potentially alleviating stress on ligamentous and cartilaginous structures.

This article significantly advances the discourse on RA treatment by proposing an innovative therapeutic rationale using manual therapy and therapeutic exercise. However, it's pivotal to acknowledge the theoretical nature of the proposed protocol; empirical validation through controlled clinical trials is imperative to ascertain its clinical efficacy and superiority compared to existing treatments. It would be also appropriate to carry out electromyographic studies to verify the actual muscle activations during the proposed treatments. Additionally, future research avenues could explore comparative studies among various manual therapy techniques and the development of specific, evidence-based therapeutic exercises targeting TMj rehabilitation.

## Conclusions

This article underscores the pressing need for refined treatment modalities for RA. This novel approach offers a specific and biomechanically grounded protocol for the management of RA. By addressing the critical balance between passive ligament structures and active muscle in the TMj, this protocol aims to enhance joint stability, reduce pain, and improve functional outcomes for RA patients. The lack of comparative studies between manual therapy techniques and the specificity of therapeutic exercises in previous approaches necessitates further clinical validation of this proposed protocol. The targeted focus on the 1st DI muscle as a key abductor provides a promising avenue for managing this debilitating condition more effectively.
